# Epidemiology of antenatal depression in Africa: a systematic review and meta-analysis

**DOI:** 10.1186/s12884-020-02929-5

**Published:** 2020-04-28

**Authors:** Abel Fekadu Dadi, Haileab Fekadu Wolde, Adhanom Gebreegziabher Baraki, Temesgen Yihunie Akalu

**Affiliations:** 1 University of Gondar, College of Medicine and Health Sciences, Institute of Public Health, Department of Epidemiology and Biostatistics, Gondar, Ethiopia; 2 School of Public Health, College of Medicine and Public Health, Flinders University, Adelaide, Australia

**Keywords:** Antenatal depression, Associated factors, Systematic review, Meta-analysis, Africa

## Abstract

**Background:**

Antenatal depression is a serious problem worldwide that has devastating consequences not only for the mother but also for the child and family. The pooled evidence regarding the prevalence and associated factors of antenatal depression is rare in Africa. Hence this review aimed to investigate the prevalence and associated factors of antenatal depression in Africa.

**Methods:**

We searched CINHAL, MEDLINE, PsycINFO, Psychiatry online, PubMed, SCOPES, and Emcare databases for English written observational studies conducted in Africa from 2007 to 2018.Quality of studies was assessed using the Newcastle Ottawa Scale (NOS), and studies with good quality were included in the final review. Heterogeneity across studies was assessed using the *I*^*2*^ and Higgins test. Publication bias was checked using Funnel plot symmetry, and Egger’s regression test and adjustment was made by using Duval and Tweedie’s Trim and Fill analysis. A random effect Meta-analysis was employed to determine the pooled estimates with 95% confidence interval (CI). Stata 14 was used for analysis. The review protocol has been registered in PROSPERO number CRD42018106717.

**Result:**

Of the 175 studies identified, 28 studies with an overall sample size of 17,938 were included. According to the random effect model following trim and fill analysis, the pooled prevalence of antenatal depression in Africa was 26.3% (95%CI: 22.2, 30.4%). Economic difficulties [POR = 1.87;95%CI:1.25,2.78,*I*^*2*^ = 88.1%], unfavorable marital condition [POR = 4.17;95% CI:1.75, 9.94, *I*^*2*^ = 81.2%], poor support from relatives [POR = 1.36;95% CI:1.18, 1.56, *I*^*2*^ = 78.0%], bad obstetric history [POR = 2.30;95% CI:1.81, 2.92), *I*^*2*^ = 81.7%], and history of mental health problem [POR = 2.97; 95% CI:1.74, 5.06, *I*^*2*^ = 92.0%]were the factors associated with antenatal depression.

**Conclusion:**

The prevalence of antenatal depression is high in Africa, which showed that one in four pregnant women had depression. Pregnant mothers who had economic difficulties, bad obstetric history, poor support from relatives, previous mental health problems, and unfavorable marital conditions were at higher risk of antenatal depression. Therefore these factors should be considered while designing mental health care services for pregnant mothers.

## Background

Depression is one of the types of mood disorders characterized by markedly decreased interest or pleasure in almost all activities, significant weight loss or gain, disturbed sleep, feeling of fatigue, loss of appetite, feeling of hopelessness, reduced self-esteem and confidence, diminished ability to think or concentrate, and recurrent thoughts of death [1, 2]. According to the World Health Organization (WHO) 2017 estimate, 322 million people are living with depression, and29.9 million (9%) of these are living in Africa. Depressive disorders are ranked among the top five contributors to the global disease burden [3]. The prevalence of depression increased by 18.4% between 2005 and 2015 worldwide [4].

Antenatal depression is a non-psychotic depressive episode ranging from mild to severe symptoms that occur while the woman is pregnant [5, 6]. Women are known to be at higher risk of mental disorders like depression than males [7]. The mental health of women of reproductive age is becoming a significant public health problem both in developing and developed countries, and depression is the most prevalent mental disorder during pregnancy [8, 9]. A systematic review and meta-analysis conducted in developed countries showed the prevalence of depression to be 7.4, 12.8, and 12% at the first, second, and third trimesters of pregnancy, respectively [10]. Another meta-analysis conducted worldwide also reported the prevalence of antenatal depression that ranges from 0.5 to 51% [11]. Moreover, a similar study from low-and middle-income countries also showed a mean weighted prevalence of common mental disorders during pregnancy to be 15.6% [12]. The magnitude of antenatal depression varies across different countries in Africa, and studies showed the prevalence to be between 8.3 and 78.2% [8, 13–18].

Depressive disorders during pregnancy may have devastating consequences not only for the mother but also for the child and family [19]. Antenatal depression is identified to be a risk factor for adverse obstetric and birth outcomes like fetal growth retardation, low Apgar score, preterm birth, low birth weight, and stillbirth [13, 20–23]. Antenatal depression is also associated with increased smoking, alcohol consumption, and unhealthy behaviors [24]. These factors, together with depression, may predispose the mothers to obstetric complications [19] such as preterm labor [25], preeclampsia, abruption placenta [26, 27]. Furthermore, depression during pregnancy is also associated with postnatal depression [28], which negatively affects child development, mother-infant interaction, and the family at large [9].

Compared with women in developed countries, women in developing countries are more exposed to the risk factors for the development of antenatal depression, such as; younger age of mothers [18], low level of education, exposure to domestic violence [8] or relationship conflicts [18], history of obstetric complications, history of depression [15, 29, 30], unplanned pregnancy, lack of social support [27, 31], and low economic status [30].

Despite variations in the magnitude and associated factors of antenatal depression across different countries of Africa, pooling the available evidence and reporting the extent of the problem in a more precise way might help policymakers to prioritize the problem more than ever. Therefore, the objective of the current review is to assess the epidemiology of antenatal depression in Africa.

## Method

### Search strategy and selection criteria

The study conforms to the Preferred Reporting Items for Systematic Reviews and Meta-Analysis (PRISMA) guidelines [32]. The reviewed articles were sourced from the following databases: MEDLINE (via Ovid), PsycINFO, CINAHL (EBSCO), Psychiatry Online, Emcare, PubMed, Scopus. Besides, google scholar, snowballing, and retrieving references from a list of eligible studies were employed. A search strategy was developed for each database by using a combination of free texts and controlled vocabularies (i.e., Mesh terms).

Example of the search strategy for PubMed:

((Prenatal depression) OR antenatal depression) OR "depression during pregnancy" Filters: Observational Study; Publication date from 2007/01/01 to 2018/08/02; Humans; English; Africa

We included all observational (cross-sectional, case-control, prospective, and retrospective cohort) studies conducted in Africa, which conducted from 2007 up to 2018 and included antenatal depression and associated factors as a primary outcome. Additionally, studies included must have used a validated tool to screen depression, and they must be available in the English language. Studies that were reviewed, studies with a poor quality based on NOS [33],and studies that were conducted on high-risk population groups were excluded from the review.

Articles were independently screened in two stages: firstly, the titles and abstracts were screened, and secondly, the full-text articles that met the eligibility criteria mentioned above were retrieved and screened further for possible inclusion by two reviewers (AFD and HFW). Where there was disagreement between the two reviewers, further discussion was made until a consensus reached.

### Data extraction

The data extraction sheet was prepared to collect information on the name of the author, year of publication, country, study setting(i.e., population-based versus institution-based), study design, sample size, time of screening for the depression, the tool used to screen depression, and the prevalence estimates of antenatal depression. Data were extracted by two reviewers (AFD and AGB) from publications, and HFW crosschecked for accuracy.

### Data quality and risk of bias assessment

The quality of evidence and risk of bias for studies (case-control and cohort) was assessed using the Newcastle-Ottawa Scale (NOS). Crossectional studies were also assessed using an adapted version of NOS. The criteria include 3 categories with a maximum score of 9 points. The first is the “selection” category, which accounts for a maximum of 4 points, the second is the “comparability” category, which accounts for a maximum of 2 points, and the third is “outcome,” which accounts for a maximum of 3 points. Based on the composite score from these three categories, the studies were classified as good quality if the score is ≥7 points, fair quality 2 to 6 points, and poor quality ≤1 point [33]. Only studies with good quality were included in this review.

### Data analysis

Meta-analysis was conducted to synthesize the pooled prevalence of antenatal depression and the odds ratio of the associated factors using the random effect model. To determine the extent of variation between the studies, we did a heterogeneity test using the Higgins method, which was quantified by *I*^*2*^ values [34]. Publication bias was assessed by using the symmetry of the Funnel plot and statistically by Egger’s regression test. Duval and Tweedie’s Trim and fill analysis was done to correct for publication bias [34, 35]. Sub-group analysis for the pooled prevalence was done, and sensitivity analysis was also carried out to detect any effect of outlier study that significantly affected the pooled estimate. Odds ratio with a 95% confidence interval was used to assess the association between antenatal depression and associated factors. The analysis was done using Stata version 14 software [36].

### Protocol registration

The review protocol has been registered in PROSPERO with protocol number CRD42018106717.

## Result

### Search

**Fig. 1:**
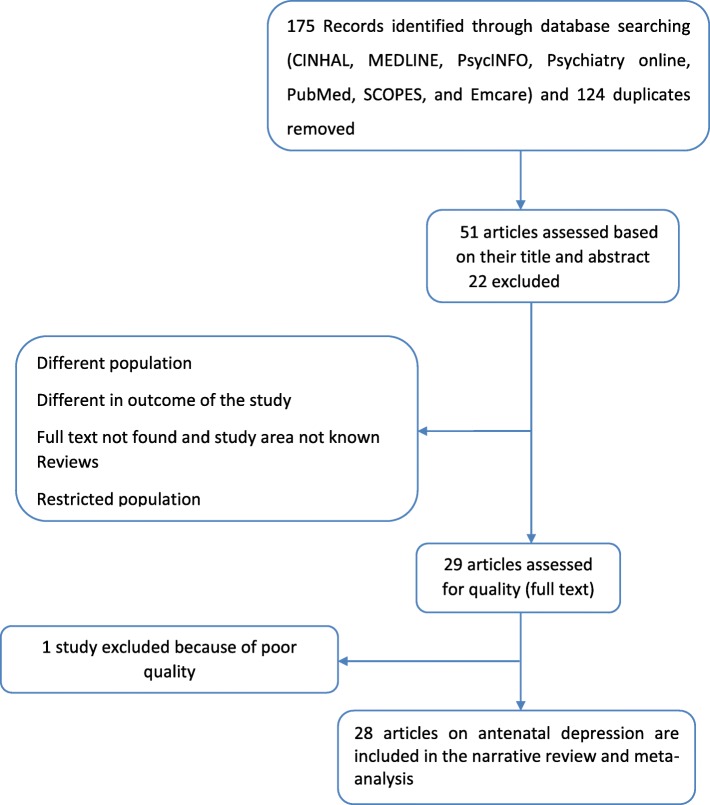
PRISMA statement presentation for systematic review and meta-analysis of antenatal depression in African countries

We conducted an electronic literature search and identified 175 unique records of journal articles and 124 duplicates that were removed. After meticulous review of the titles and abstracts, we excluded 22 articles because of difference in population under study [37–42] and outcome of the study [43–45], full text not found and study area not known [46–50], reviews [5, 19, 51–53] and conducted in restricted population [54, 55]. We obtained full-text copies of 29 records for further review. Of those full-text articles, one article [56] was excluded because of poor quality. Finally, 28 articles with an overall sample size of 17, 938 were included in the narrative review and Meta-analysis (Fig. 1).

### Included study characteristics

The sample size across the studies ranges from 109 [57] to 2086 [58] pregnant mothers. The selected studies for Meta-analysis were geographically diverse and included 10 African countries with the majority (9 studies) of the studies from Ethiopia [17, 27, 30, 31, 45, 59–62] followed by South Africa, which contains five studies [18, 57, 63–65]. In terms of the study setting majority of the included studies [20] were health institution based [8, 13, 15, 16, 27, 57, 59–64, 66–72] and the rest were community-based [17, 18, 30, 31, 45, 58, 65, 73]. Moreover, 21 studies used cross-sectional [8, 15–18, 27, 30, 31, 57, 59–64, 66–70, 73], six studies used cohort [13, 45, 58, 71, 72], and one study used longitudinal study design [65].

The included studies used different tools for screening depression. The tool used by the majority [11] of the studies was EPDS at a different cut off value [18, 27, 30, 31, 62, 63, 65, 69, 70, 72, 73] followed by PHQ [5] [13, 17, 45, 71]. Most of the included studies [13] screened depression in all trimesters of pregnancy [8, 16, 18, 27, 30, 59, 60, 62, 64–66, 69, 70] followed by screening in the third trimester of pregnancy, which was applied in 8 studies [13, 15, 31, 61, 63, 71, 73]. The prevalence of depression among the included studies ranges from 8.3% [15] to 78% [16] and all of the included studies have high quality based on NOS (Table 1).

**Table 1:** Summary of studies conducted on antenatal depression in African countries (*N* = 64, 2007–2018)

Author, P. year	Country	Study setting	Study design	Sample size	Time of screening	The tool used for screening depression	Prevalence	Final score of NOS assessment
Adewuya, A. O. et al. 2007	Nigeria	HI	cross-sectional	180	Third trim	DSM-IV	8.30%	7
Esimai, O. et al. 2008	Nigeria	HI	cross sectional	195	All trim	HADS (not found)	10.80%	7
Kaaya SF et al. 2010	Tanzania	HI	cross sectional	560	Second trim	HSC ≥ 1.06	39.50%	7
Hartley M et al., 2011	South Africa	Community	cross-sectional	1062	All trim	EPDS ≥14	39%	8
Rochat TG et al., 2011	South Africa	HI	cross-sectional	109	Second trim	DSM-IV	47%	7
Manikkam L et al., 2012	South Africa	HI	cross-sectional	387	Third trim	EPDS ≥13	38.50%	7
Stewart RS et al., 2014	Malawi	HI	cross-sectional	583	Second trim	SRQ ≥ 8	21.10%	7
Weobong B et al. 2014	Ghana	Community	cohort	2086	First trim	PHQ ≥ 10	9.90%	8
Abdelhai R et al. 2015	Egypt	HI	cross-sectional	376	All trim	HADS> 10	10.40%	8
Bindt C et al. 2013	Ghana	HI	cohort	719	Third trim	PHQ ≥ 10	28.90%	8
Mahenge B et al. 2015	Tanzania	HI	cross sectional	1180	All trim	HSC ≥ 1.06	78.20%	7
RwakaremaM et a; 2015	Tanzania	HI	cross-sectional	397	All trim	EPDS ≥13	33.80%	8
Heyningen T et al. 2015	South Africa	HI	cross-sectional	376	All trim	MINI	22%	7
Malqvist M et al. 2016	Swaziland	Community	cross-sectional	1038	Third trim	EPDS ≥13	22.70%	7
Thompson O et al. 2016	Nigeria	HI	cross-sectional	314	All trim	EPDS> 11	24.50%	8
Dibaba Y et al. 2013	Ethiopia	Community	cross-sectional	627	Third trim	EPDS ≥13	19.90%	8
Gemta A et al. 2013	Ethiopia	HI	cross-sectional	660	All trim	EPDS (not found)	25.60%	8
Biratu A et al. 2015	Ethiopia	HI	cross-sectional	393	All trim	EPDS ≥13	24.94%	8
Ayele TA et al. 2016	Ethiopia	HI	cross-sectional	388	All trim	BDI ≥ 16	23.00%	8
Bisetegn TA et al. 2016	Ethiopia	Community	cross-sectional	527	All trim	EPDS ≥12	11.80%	8
Bitew T et al. 2016	Ethiopia	Community	cross-sectional	1311	Second trim	PHQ ≥ 5	29.50%	8
Mossie Tb et al. 2017	Ethiopia	HI	cross-sectional	196	All trim	BDI ≥ 14	31.10%	8
Sahile MA et al. 2017	Ethiopia	HI	cross-sectional	231	Third trim	BDI ≥ 21	31.20%	8
Guo N et al. 2013	Ghana	HI	cohort	654	Third trim	PHQ ≥ 10	26.30%	7
Guo N et al. 2013	Cote devour	HI	cohort	654	Third trim	PHQ ≥ 10	28.30%	7
Bitew T et al. 2017	Ethiopia	Community	cohort	1240	2nd & third	PHQ ≥ 5	28.70%	8
Mochache K et al. 2018	Kenya	HI	cohort	255		EPDS ≥10	38.40%	8
Thai A et al. 2016	South Africa	Community	longitudinal	1238	All trim	EPDS> = 13	39.50%	7

*CIS-R* Clinical Interview Schedule–Revised, *HSC* Hopkins symptom checklist, *EPDS* Edinburgh Postnatal Depression scale

### Pooled prevalence of antenatal depression

**Fig. 2:**
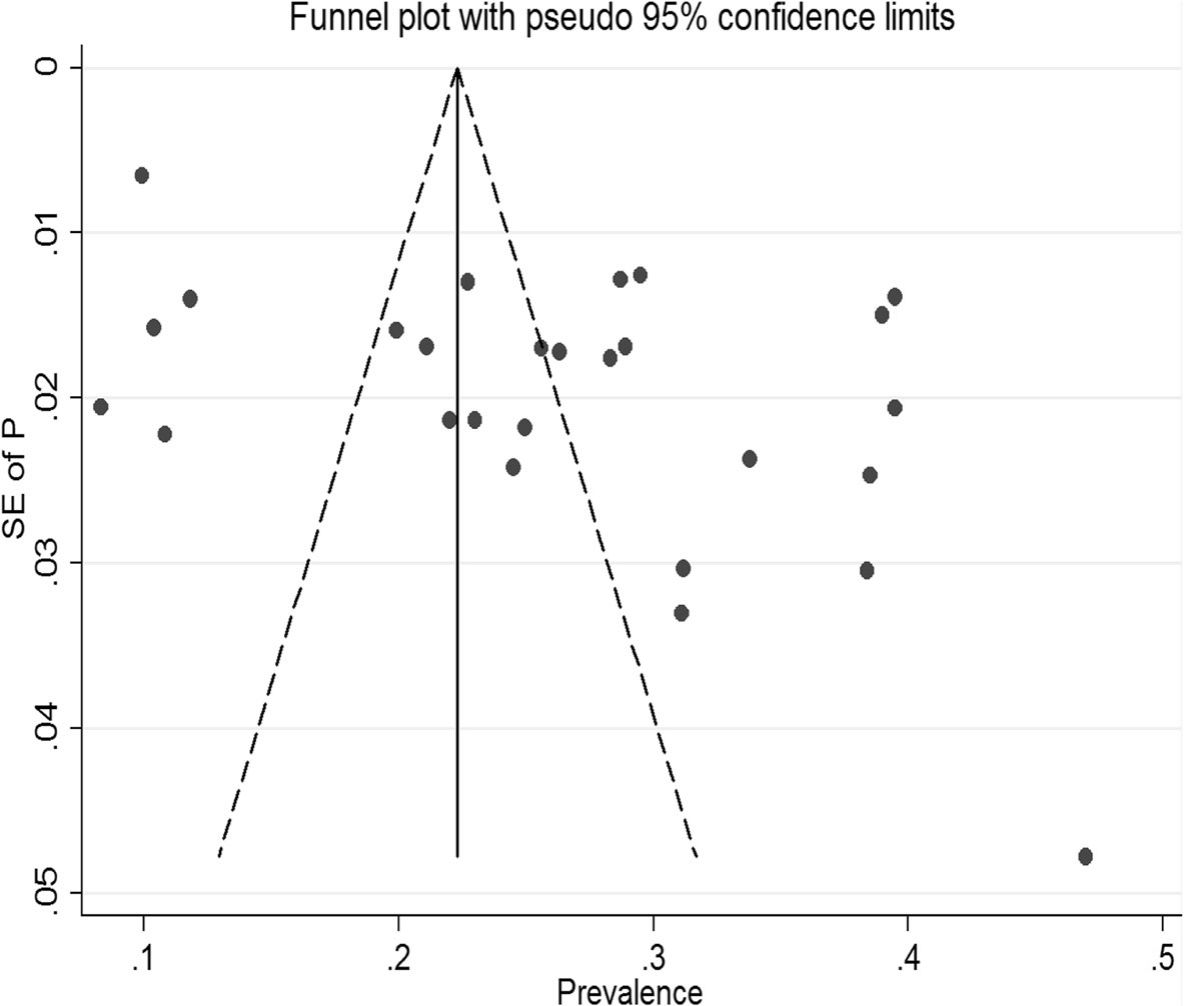
Funnel plot testing publication bias (random, *N* = 27)

**Fig. 3:**
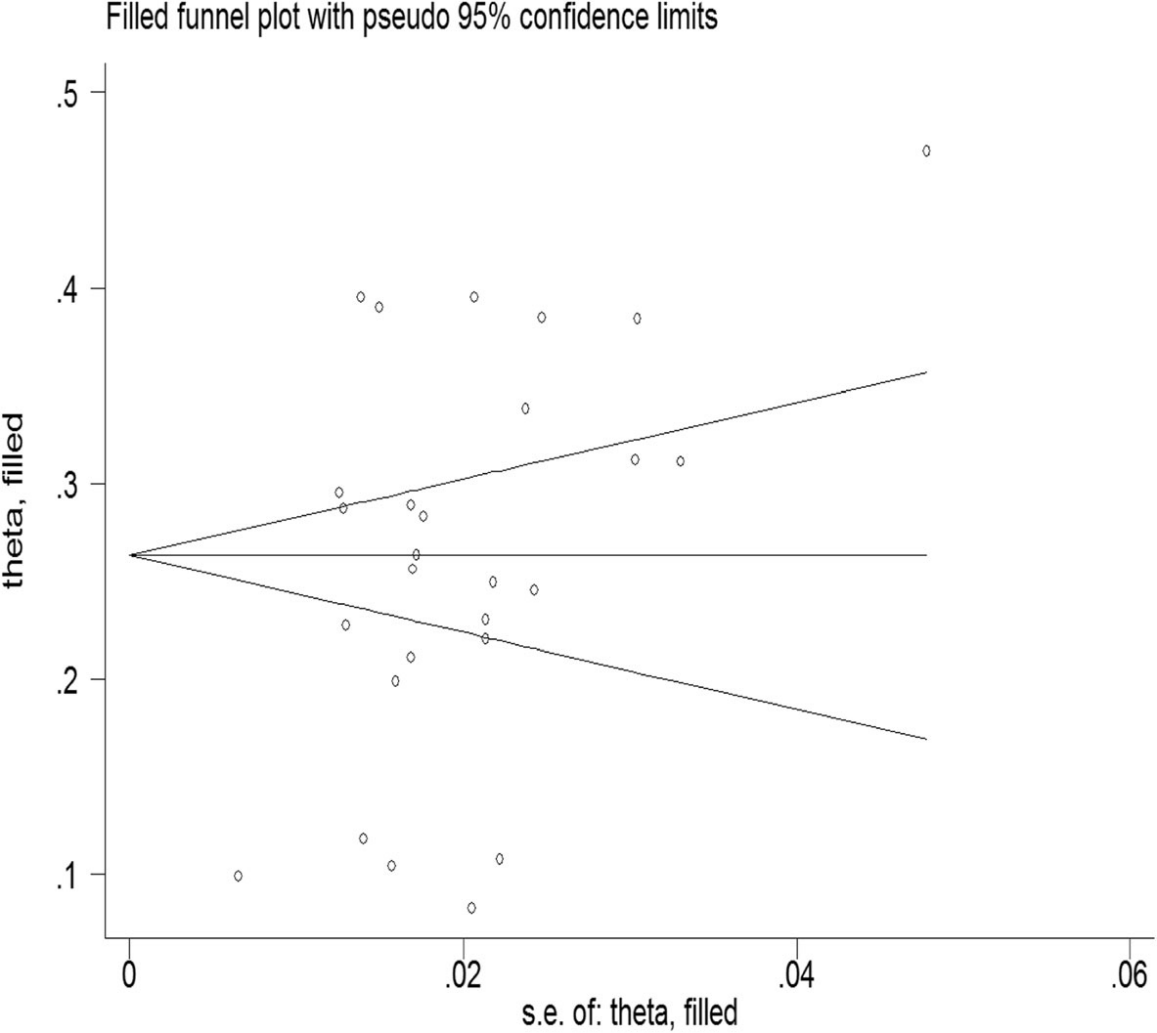
Filled funnel plot after adjusting for publication bias

**Fig. 4:**
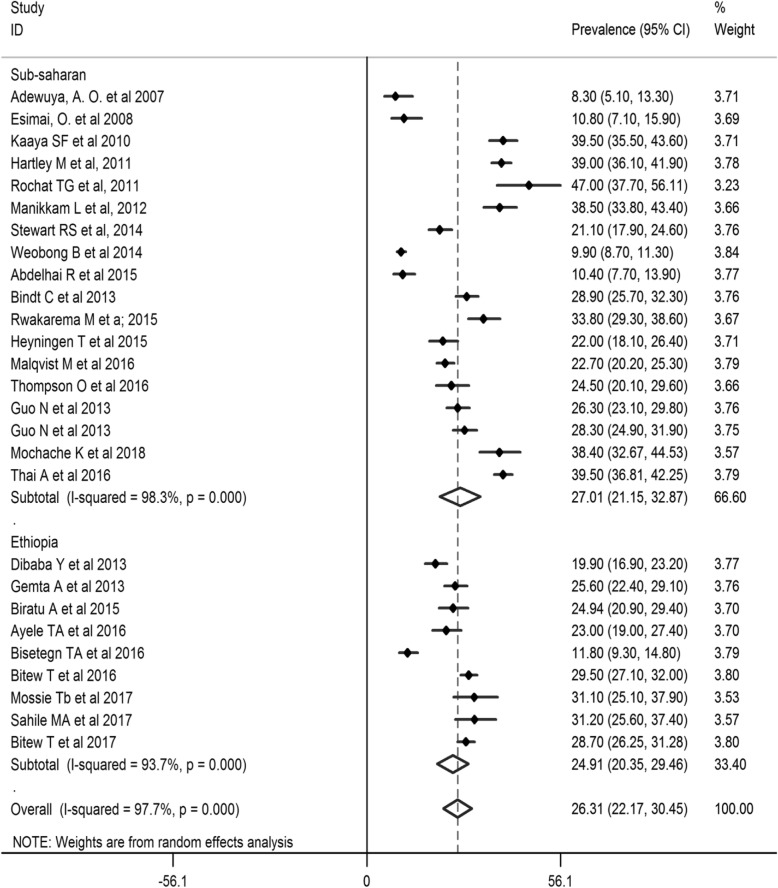
Forest plot for meta-analysis of antenatal depression prevalence sub-analyzed by geography in Africa (*N* = 28, random effect model)

The pooled prevalence of antenatal depression in Africa from 28 studies was found to be 26.3% (95% CI: 22.2, 30.4%;*I*^*2*=^ 97.7%).As eggers test was found significant, the final pooled prevalence was corrected for Duval and Tweedie’s trim and fill analysis and was 26.3% (95%CI; 22.2, 30.4%) (Figs. 2 and 3). The meta-regression was conducted to identify study characteristics accounted for heterogeneity. The pooled prevalence was higher at 27.01% (95% CI: 21.15, 32.87%)in Sub Saharan Africa (SSA) than the pooled rate in Ethiopia at 24.91% (95% CI: 20.35, 29.46%) (Fig. 4).

Besides, subgroup analysis was done based on the income of countries, time of depression measurement, study setting, study design, year of publication, and tool used to screen depression. Based on the time of screening for depression in the course of pregnancy, the highest pooled prevalence of antenatal depression was observed in the second trimester, 32.20% (95% CI: 26.13, 38.28%). On the other hand, a significantly lowest prevalence of was found from a single study with a screening time in the first trimester, 9.90% (95% CI: 8.60, 11.20%). Depending on the study setting, the pooled prevalence of antenatal depression for 19 health institution based studies was 26.77% (95%: 22.54, 30.99%).

Based on the study design, the pooled prevalence of antenatal depression for seven studies, which applied longitudinal study design was higher, at 28.49% (95% CI: 18.47, 38.52%), as compared to those using cross-sectional study designs. In terms of the sample size, the pooled prevalence of antenatal depression for 19 studies with a sample size above 384 was 27.43% (95% CI: 22.54, 32.31%). Concerning the year of publication, the highest pooled prevalence was observed from 4 studies published between 2010 and 2012, 39.44% (95% CI: 37.37, 41.50%). On the other hand, the lowest pooled prevalence of 9.46% (95% CI: 6.46%, 12.46) was observed from studies published between 2007 and 2009. Depending on the screening tool used, the pooled prevalence of antenatal depression was the highest for 11 studies that used EPDS (PP = 28.89%; 95% CI: 22.79, 34.98%) (Table 2).

**Table 2:** Sub-analysis of studies on antenatal depression conducted in Africa (*N* = 27, random effect)

Variable for sub-analysis	Number of studies	Sample size (*N*)	Pooled prevalence (95%CI) random effect model
The income of the countries			
Low-income	12	7115	26.54(22.23, 30.85)
Middle-income	15	7775	26.13 (19.60, 32.64)
Time of depression measurement			
1st trimester	1	2086	9.90 (8.60, 11.20)
2nd trimester	5	3803	32.20 (26.13, 38.28)
3rd trimester	8	4312	25.39 (20.15, 30.65)
All trimester	13	4689	25.71 (19.30, 32.11)
Study setting			
Community-based	8	7891	25.11 (16.52, 33.69)
Health institution based	19	6999	26.77 (22.54, 30.99)
Study design			
Longitudinal	7	5353	28.49 (18.47, 38.52)
Cross sectional	20	9537	25.50 (21.16, 29.84)
Sample size			
< =384	8	1626	23.59 (15.49, 31.69)
> 384	19	14,757	27.43 (22.54, 32.31)
Year of Publication			
2007–2009	2	375	9.46 (6.46, 12.46)
2010–2012	4	2118	39.44 (37.37, 41.50)
2013–2015	11	7525	22.75 (17.32, 28.18)
2016–2018	10	6740	27.92 (22.41, 33.43)
Type of screening tool used			
EPDS	11	6898	28.89 (22.79, 34.98)
PHQ-9	5	4578	28.54 (27.23, 29.84)
Diagnostic tools (DSM-IV,CIS-R)	3	665	25.22 (8.33, 42.11)
Other (CES-D, SRQ, HSC, BDI)	8	4617	21.96 (14.30, 29.62)

**Fig. 5:**
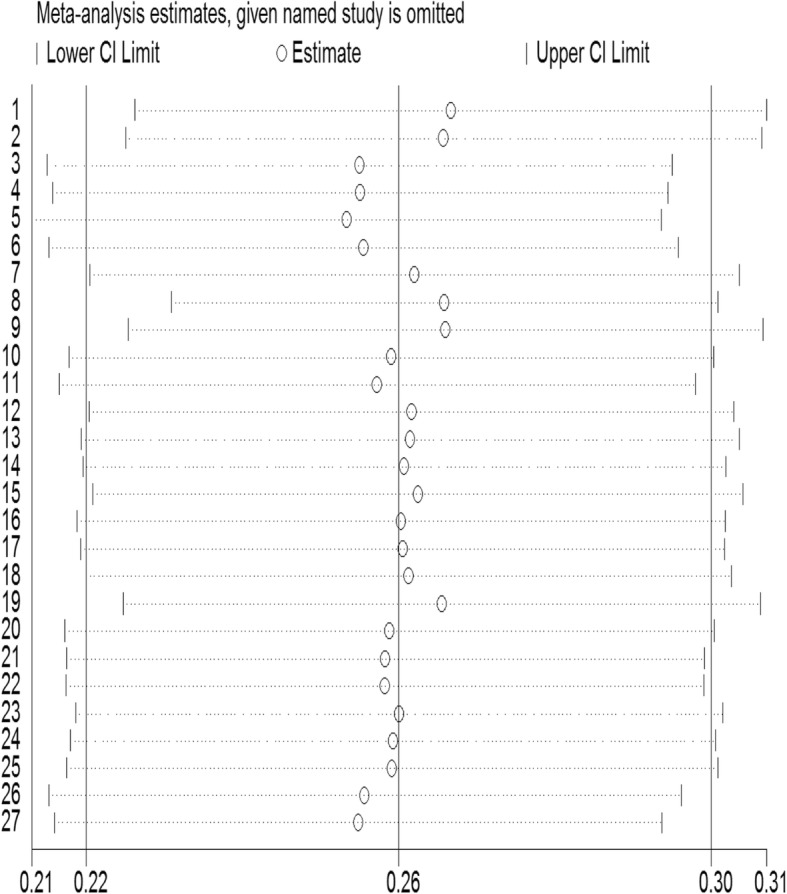
Sensitivity analysis for studies included in the meta-analysis

Sensitivity analysis was completed after excluding the study with the highest prevalence [16], and it showed that omission of any of the incorporated studies did not change the pooled prevalence of antenatal depression (Fig. 5).

**Fig. 6:**
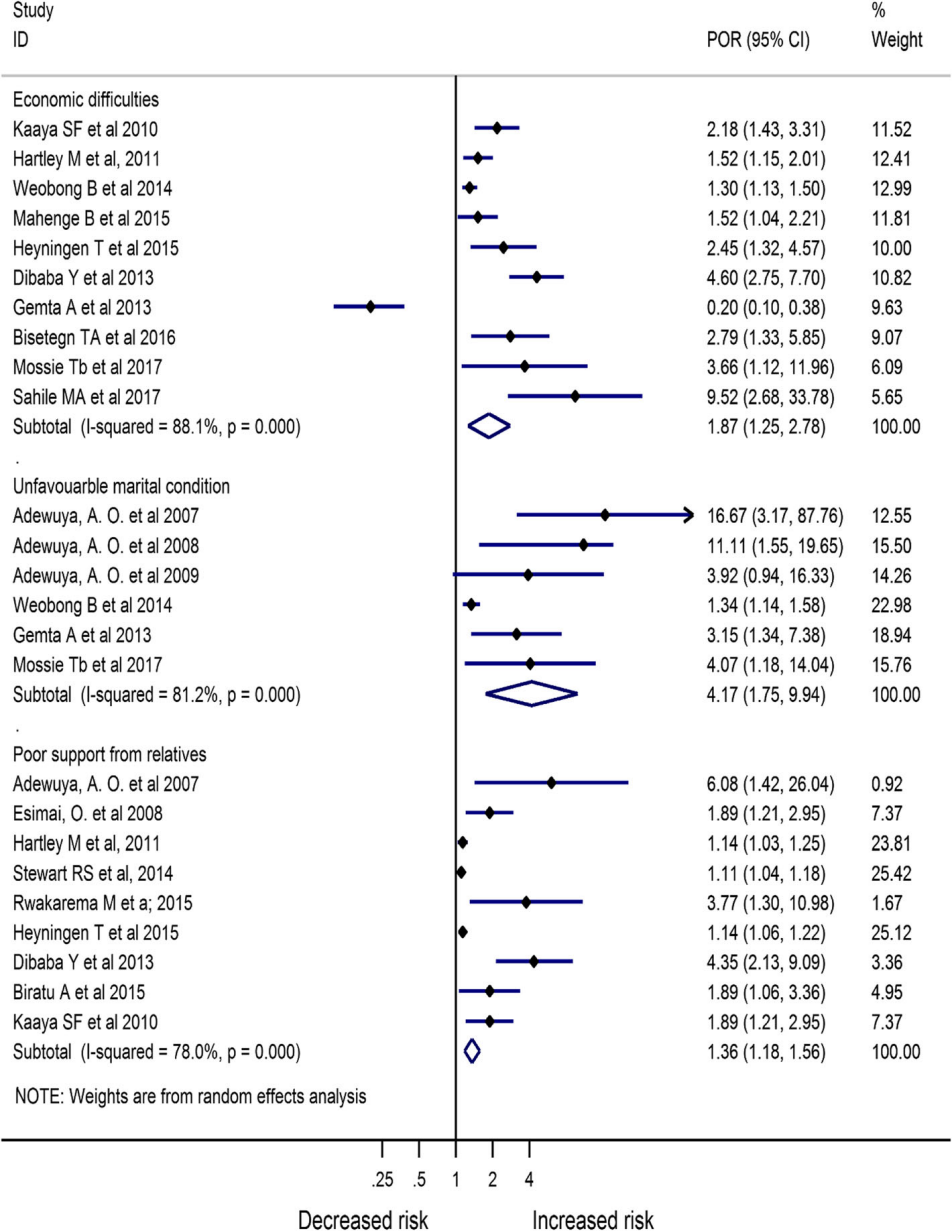
Meta-analysis of major risk factors associated with antenatal depression in Africa

**Fig. 7:**
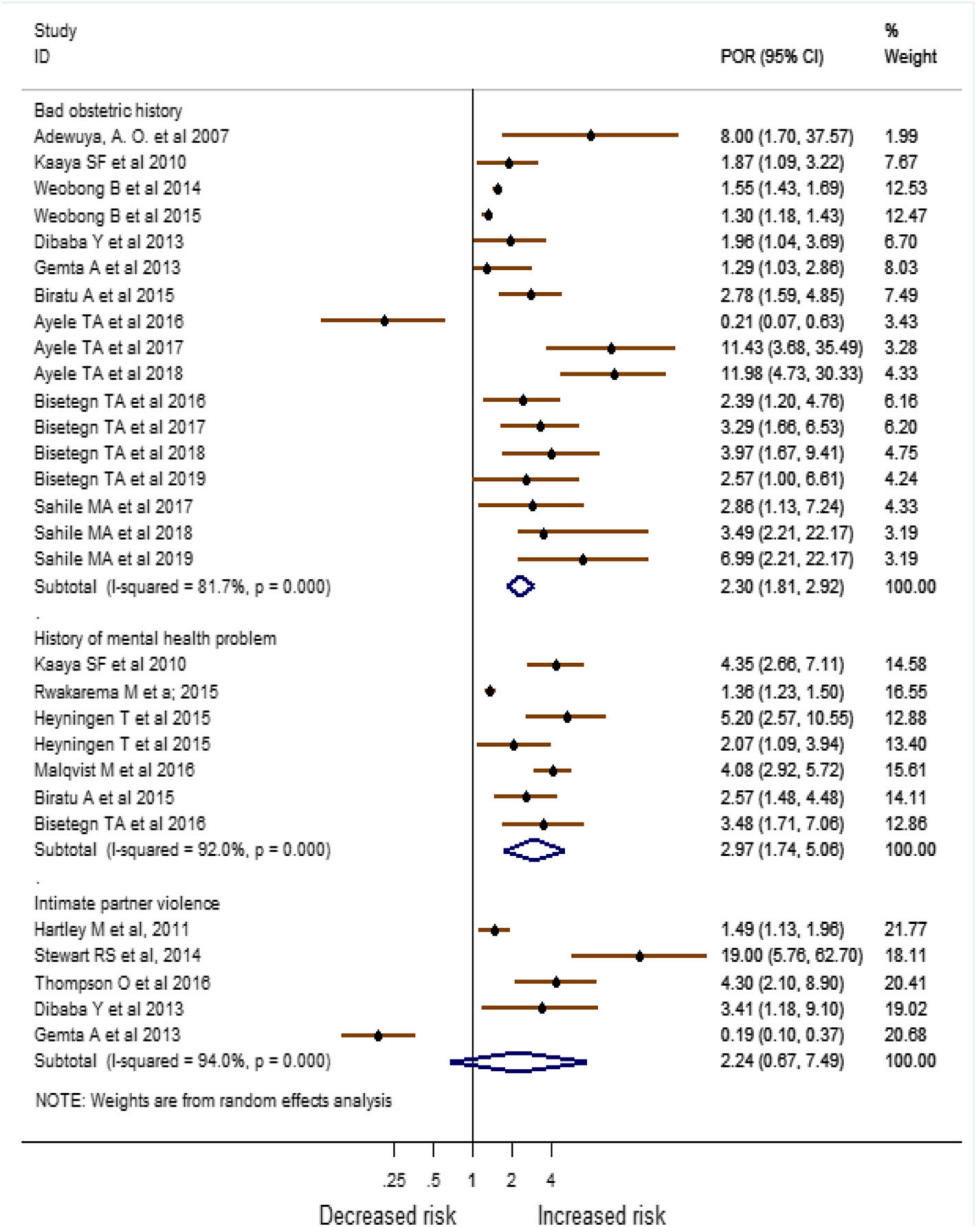
Meta-analysis of major risk factors associated with antenatal depression in Africa

Based on the result from Meta-analysis of identified risk factors, economic difficulties [POR = 1.87 (95% CI:1.25, 2.78), *I*^*2*^ = 88.1%], unfavorable marital condition [POR = 4.17 (95% CI:1.75, 9.94), *I*^*2*^ = 81.2%], poor support from relatives [POR = 1.36 (95% CI:1.18, 1.56), I^2^ = 78.0%], bad obstetric history [POR = 2.30 (95% CI:1.81, 2.92), I^2^ = 81.7%], and having history of mental health problem [POR = 2.97 (95% CI:1.74, 5.06), I^2^ = 92.0%] were the major factors associated with antenatal depression (Figs. 6 and 7).

## Discussion

The current review assessed the prevalence of antenatal depression and its associated factors in Africa. Our review showed the pooled prevalence of depression among African pregnant women was 26.3%, and it is significantly associated with economic difficulties, poor support from relatives, bad obstetric history (such as previous pregnancy loss and complications), unfavorable marital condition, and history of mental health problems.

The result was consistent with a review conducted in low-and middle-income countries, which showed a pooled prevalence of 25.3% [74]. The pooled prevalence of antenatal depression from this review was found to be higher than other reviews done in Africa and low-and middle-income countries, which showed that the weighted mean prevalence of common mental health disorders to be 11.3 and 15.6%, respectively [12, 75]. This might indicate that antenatal depression is increasing over time. Our estimate was also higher than another systematic review and meta-analysis done in developed countries, which showed the pooled prevalence at the first, second, and third trimesters to be 7.4, 12.8, and 12%, respectively [10]. The higher pooled prevalence in our review could be mothers in Africa are exposed to additional socio-economic problems and stressful life events than mothers from developed countries, which increases their risk of developing depression.

The odds of having antenatal depression among women who have economic difficulties was found to be 1.87. This result was consistent with other systematic reviews published in Ethiopia, low-and middle-income countries, and worldwide [5, 12, 76, 77]. The finding was also supported by another large scale prospective cohort study conducted to assess the association between low socioeconomic status and antenatal depression [78]. Mothers in such economic difficulties would worry about the family and the coming babies’ basic needs, and this can also be related to family food insecurity, which is highly associated with antenatal depression [31, 79].

This review showed that unfavorable marital condition (explained as divorce, relationship difficulties, being single at the time of pregnancy, marital conflict) was significantly associated with increased risk of antenatal depression by4.17 times. A similar association was found from other systematic reviews done in Ethiopia and other low-and middle-income countries [5, 12]. This might be because of a lack of support from husband at the time of pregnancy as those women who receive husbands’ support during their pregnancy may be well empowered to deal with their pregnancy and their home responsibility. Another reason could be those women with unfavorable marital conditions might live alone, practice more loneliness, and low self-confidence that may predispose them to depression.

This review also showed that there was an increased risk of antenatal depression among women who had poor support from relatives, which is supported by other previous reviews [12, 76, 80]. The possible reason could be because social support reduces stressful life events by providing informational, instrumental, and emotional support during pregnancy [80, 81]. Perhaps, the objective evaluation of social support that women received during pregnancy may be challenging as it has been noticed that depressed women tend to feel less supported than they objectively are [82]. Women with a bad obstetric history were found to be at an increased risk of antenatal depression by an odds of 2.30 times. This result was similar to other reviews done in Ethiopia [5, 77] and worldwide [80]. This is directly associated with the mother’s fear of facing similar complications in her current pregnancy like the previous one.

Women with a history of mental health problems also had three-fold increased odds of developing antenatal depression in the current pregnancy as compared to those who did not report such history. This result was supported by other reviews done in Ethiopia [5, 77] and worldwide [80]. Having a history of mental disorders, including depressive episodes, may indicate the mother’s biological vulnerability to the disorder, which may indirectly cause pregnancy mood changes in the current pregnancy [83].

The strength of this review is that it included only high-quality studies that scored ≥ seven based on the NOS criteria, and this may increase the reliability and validity of the findings. Besides, all available observational studies in Africa that satisfied our inclusion criteria were included, and this would increase the review generalizability. However, the use of screening instruments in prevalence studies relies on symptom identification rather than diagnosis, and this affects the validity of the review as screening tools over or underestimate the actual effect estimate. Though a subgroup and random-effect meta-analysis were conducted to minimize the effect of heterogeneity, the prevalence estimates might still be affected by the inherent heterogeneity of included studies, which may be due to the difference in the study area, methodology, study period and the type of screening tool used in the studies. Therefore, policymakers should make the interpretations of these results with caution by considering this inherent heterogeneity. Further standardization of screening tool that would be used in different countries might help to minimize the observed heterogeneity. Moreover, excluding works of literature that are not published in the English language may create selection bias. The clinical importance of this review is that it clearly showed that depression during pregnancy is highly prevalent and needs to be targeted for screening and early treatment.

## Conclusion

We found that one in four mothers had antenatal depression, and it is independently associated with a mother’s economic difficulties, poor support from relatives, unfavorable marital condition, previous history of mental disorder, and bad obstetric history. Depression screening during pregnancy follow up in African health facilities is highly relevant to early detect and intervene in women’s at risk of antenatal depression. This would help the achievement of sustainable development goal five (SDG-5) through improving maternal mental health.

## Data Availability

All the materials and data on which the findings of this review are based are presented within the manuscript.

## Abbreviations

BDI: Beck Depression Inventory
CI: Confidence Interval
DSM-IV: Diagnostic and Statistical Manual of mental disorders version 4
EPDS: Edinburgh Postnatal Depression Scale
HADS: Hospital Anxiety and Depression Scale
LAMICs: Low And Middle-Income Countries
NOS: Newcastle Ottawa Scale
PHQ: Patient Health Questionnaire 9
POR: Pooled Odds Ratio
PRISMA: Reporting Items for Systematic reviews and Meta-Analysis
SDG-5: Sustainable Development Goal 5
SSA: Sub Saharan Africa
WHO: World Health Organization
